# Phycosynthesis and Enhanced Photocatalytic Activity of Zinc Oxide Nanoparticles Toward Organosulfur Pollutants

**DOI:** 10.1038/s41598-019-43368-3

**Published:** 2019-05-03

**Authors:** Tariq Khalafi, Foad Buazar, Kamal Ghanemi

**Affiliations:** Department of Marine Chemistry, Khorramshahr University of Marine Science and Technology, P.O. Box 669, Khorramshahr, Iran

**Keywords:** Physical sciences, Biosynthesis

## Abstract

A novel eco-friendly procedure was developed to produce safer, stable and highly pure zinc oxide nanoparticles (ZnO NPs) using microalgae Chlorella extract. The ZnO NPs were synthesized simply using zinc nitrate and microalgae Chlorella extract which conducted at ambient conditions. In this recipe, microalgae Chlorella extract acted as the reducing agent and a stabilizing layer on fresh ZnO NPs. UV–visible spectrum was confirmed the formation of ZnO NPs showing an absorption peak at 362 nm. XRD results demonstrated that prepared ZnO NPs has a high-crystalline hexagonal (Wurtzite) structure, with average size about 19.44 nm in diameter. FT-IR spectral analysis indicated an active contribution of algae-derived biomolecules in zinc ions bioreduction. According to SEM and TEM observations, ZnO NPs are well dispersed and has a hexagonal shape with the average size of 20 ± 2.2 nm, respectively. Based on gas chromatography analyses, the optimum 0.01 g/L dosage of ZnO catalyst revealed an effective photocatalytic activity toward the degradation (97%) of Dibenzothiophene (DBT) contaminant as an organosulfur model in the neutral pH at the mild condition. Rapid separation and facile recyclability at five consecutive runs were demonstrated high efficiency and durability of green ZnO nanophotocatalyst. The possible mechanisms of green ZnO NPs formation and the photo-desulfurization of DBT were also proposed.

## Introduction

Green nanotechnology has been defined as the development of clean technology which proactively affects the design of nanomaterials and products by decreasing or eliminating pollution from the fabrication of the nanoscale materials^1^. It has drawn on a framework of the principles of green chemistry and green engineering to enhance the environmental sustainability of nanomaterial products^2^. This methodology triggers clean production processes such as natural sources which lead to green manufacture of nanoparticles for environmental remediation, renewable energy, water treatment, hazardous chemical substitution, and waste management applications. In this connection, green synthesis procedures hold promise to fulfill growing demands on eco-friendly nanomaterials, due to reducing toxic substances, low-cost process rates, easy to handle, and relatively high energy saving^3^. A significant number of nature-derived methods have been efficaciously utilized for various types of nanoparticles (NPs) including metal and metal oxide NPs^4^. Zinc oxide (ZnO) NPs are described as a significant semiconductor material and of great interest in their characteristic properties such as wide-bandgap, high electron mobility, and great transparency in visible range^5^. These nanoparticles demonstrate anti-corrosive, antifungal, photochemical, catalytic, electrical, antibacterial, UV filtering, and photovoltaic properties. Moreover, ZnO NPs has been used extensively in miscellaneous sectors such as medication, solar cells, automotive, textiles, cosmetic, and plastic films^6–8^.

Virtually, a great amount of ZnO NPs are effectively produced using traditional chemical and physical methods. Despite the advantage of these processes in the aspect of large scale and time productions of nanomaterials, yet there are growing global concern concerning their adverse environmental and public health impact mainly due to engagement of great deal of detrimental chemicals^9^. Accordingly, there is a crucial need to develop sustainable and eco-friendly techniques for producing green NPs^10^. A considerable number of green approaches using natural sources such as microorganisms and plant extracts for zinc nanoparticle synthesis has been suggested as promising alternatives to chemical methods^11,12^. Among a variety of biosynthetic approaches, algae extracts have attracted increasing attention particularly as a synthesis platform for various inorganic nanoparticles^13^.

**Figure 1:**
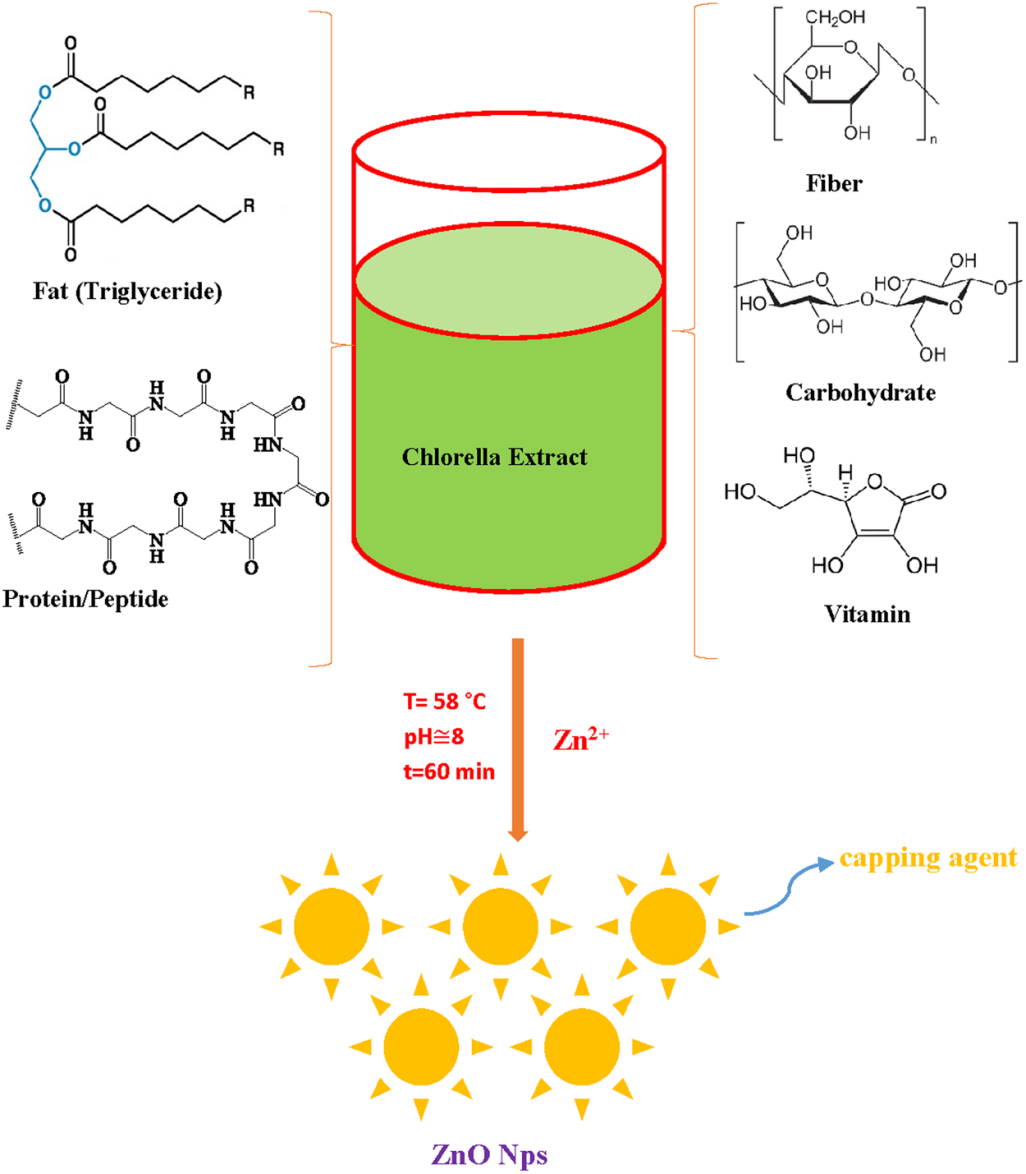
Mechanism of biosynthesis of zinc oxide nanoparticles (ZnO NPs) using algae chlorella extract as reducing and capping agent.

*Chlorella* microalgae is a biodiverse division of autotrophic organisms that typically found in aquatic environments. It form the base of the food cycle and supply energy for all higher trophic levels^14^. It is considered as a cell factory for the nanoscale particle, taking full advantages of growth rate and ample biomass productivity with a less cultivation time. Green *Chlorella* microalgae is naturally occurring biotic material that abundantly available in freshwater and marine systems. It is rich source of various organic biomacromolecules of interest (proteins, lipids, starch) which can be utilized as an alternative renewable source for the phycosynthesis of nanoparticles^15^. There are few reports of using intracellular microalgae Chlorella as an ecological medium for biosynthesis of noble metal nanoparticles including Pd, Au, and Ag^16,17^. Biosynthesis of ZnO NPs has been reported using different marine macroalgae sources including brown *Sargassum muticum*^18^, green *Caulerpa peltata*, and red *Gracilaria gracilis* with various size, shape, and morphology^19^. Hence, following our previous studies on green synthesis of NPs^9^, herein, we report a facile, green, low-cost and homemade method for preparing stable and pure ZnO NPs by microalgae Chlorella, free from chemical additives such as bases, acids and organic solvents (Fig. 1)^20^. In this work, the novel designed synthesis procedure based on microalgae platform is an environmental, economically affordable, and predominantly deter using unsafe materials which are common in conventional chemical methods^1^. Based on the literature survey, microalgae Chlorella extract is employed for the first time as a natural nanofactory for ZnO NPs biosynthesis. The morphology, phase, and structure of nanoproducts were studied by the standard characterization techniques. Lastly, the photocatalytic efficiency of bio-fabricated ZnO NPs was investigated against Dibenzothiophene (DBT) pollutant as a sulfur-containing compound model in which occurs broadly in heavier fractions of petroleum. Obviously, the ultimate disposal of DBT is extremely hazardous to the aquatic environment and shows acute toxicity to the health of living things^21^.

## Results and Discussion

### UV-visible spectral analysis

**Figure 2:**
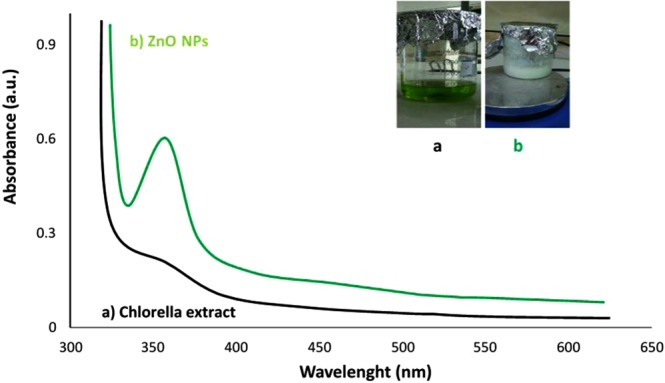
UV–visible spectra of (**a**) initial Chlorella aqueous extract and final ZnO NPs  solution (**b**); inset shows visual observations of color changes.

Bioreduction of zinc ions in watery algal medium to ZnO NPs was carried out through measuring the UV-Vis spectra of the aqueous solution in the range between 300 and 500 nm by means of UV-245 Shimadzu model spectrophotometer. The visual color change is the preliminary evidence for nanoparticle synthesis; hence, the biosynthesis process of ZnO NPs was confirmed through gradual alteration of color from light green to white precipitate (Fig. 2). UV–vis absorption spectrum of bioproduced nanoparticles demonstrated a notable peak at 362 nm (Fig. 2), which is most likely the characteristic feature of ZnO NPs. Likewise, several biological ZnO NPs synthesized by various green sources such as *Ixora Coccinea*^22^, and *Aloe vera*^23^ extracts have revealed comparable absorption peaks for bio-assisted ZnO nanoparticles.

### XRD pattern analysis

**Figure 3:**
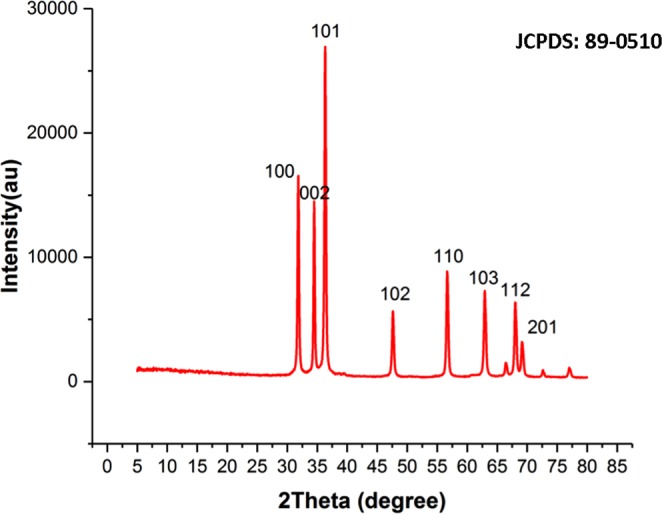
XRD pattern of the synthesized ZnO nanoparticles through Chlorella aqueous extract.

The XRD pattern provides a perception concerning the crystallinity of nanoparticle. Figure 3 represents XRD spectrum of ZnO NPs synthesized via fresh microalgae Chlorella extract. Well-defined peaks in XRD indicates that the particles were perfectly crystallized. The appearance of the prominent peaks at 31.8°, 34.47°, 36.29°, 47.56°, 56.59 °, 63.01°, 67.94° and 69.07° are attributed to the Miller-Bravais indices of (100), (002), (101), (102), (110), (311), (112) and (201), respectively. All the diffraction peaks of the ZnO nanopowder comply well with those of the Joint Committee on Powder Diffraction Standards (JCPDS, card No. 89-0510) pattern of ZnO which can be indexed to the hexagonal wurtzite crystal structure of the ZnO NPs^9,24^. Moreover, no extra peaks arising from algae extract was detected in XRD pattern presenting that ZnO NPs synthesized by the biogenic method is significantly pure. According to the most intense diffraction peak at 2*θ* = 36.29° (101), the average particle size of the produced ZnO NPs was found to be 19.44 nm using Debye–Scherrer equation^25^. This observation was further confirmed by SEM and TEM results.

### FTIR analysis

**Figure 4:**
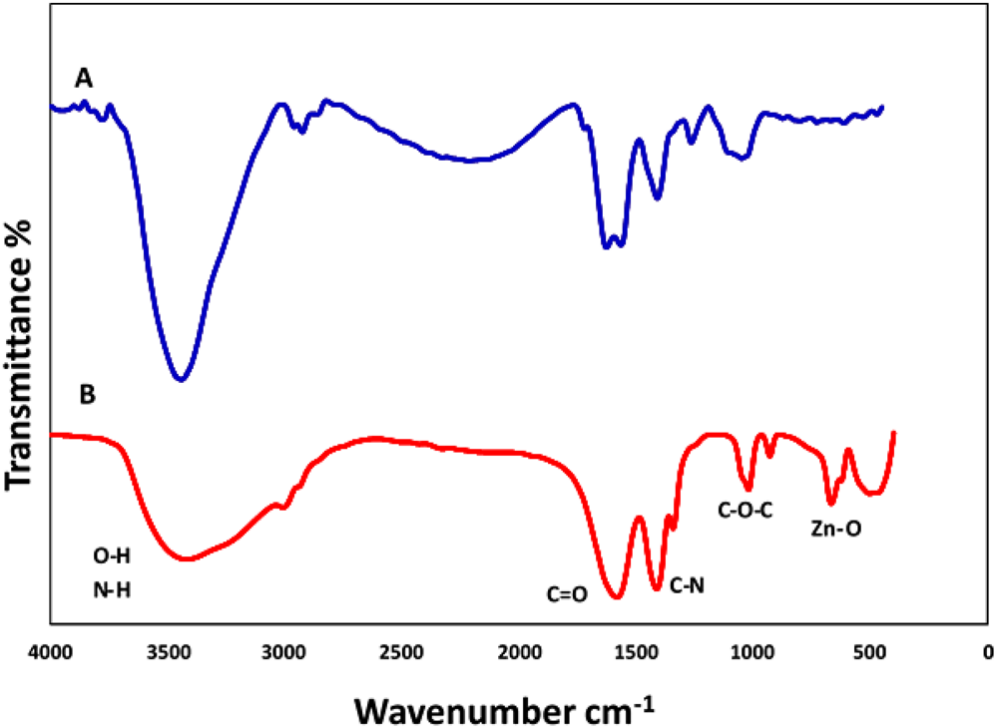
FTIR spectrum of Chlorella aqueous extract (**A**), and biosynthesized ZnO NPs (**B**).

**Figure 5:**
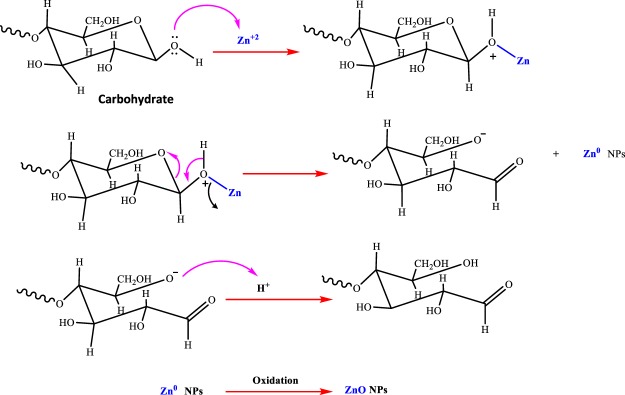
Schematic illustration of the possible mechanism for the biogenic synthesis of ZnO NPs using aqueous Chlorella extract (algae-derived carbohydrate as bioreducing agent model).

FTIR technique was performed to determine the surface chemistry of the ZnO NPs and more specifically engaged functional groups of algal extract. Generally, the FTIR spectrum relatively revealed a complex of bioorganic compounds in microalgae extract (Fig. 4A). The position of 3440, 2991, 1624, 1404 cm^−1^ bands of raw algal extract were shifted to 3415, 3000, 1600, and 1411 cm^−1^, respectively (Fig. 4B). In bio-assisted ZnO NPs, the broad peak detected at 3415 cm^−1^ can be corresponded to the protein N‒H (amide A) or O‒H stretching. Its breadth mainly resulted from inter and intramolecular hydrogen bonds formation. A short band at 3000 cm^−1^ was assigned to symmetric and asymmetric CH_2_ stretching of lipid and/or carbohydrates^26,27^ while absorption associated with the CH_2_ and CH_3_ groups of protein leads primarily to bending vibrations of the C—H bond at 1343 cm^−1^ band. A major peak around 1600 cm^−1^ is primarily due to C=O stretching vibrations of the protein amide-I bonds^28^. The absorption bands at 1411 cm^−1^ in Fig. 3 were attributed to the stretching of C—N bond of amino acid. Carbohydrate C‒O‒C ether bond of polysaccharides illustrates vibration roughly at 1021 cm^−1^. The absorption peak associated with Zn‒O stretching band clearly appears at 503 cm^−1^ confirming the formation of ZnO NPs. In literature, depending on synthesis and experimental conditions, the FTIR spectrum of several biogenic ZnO NPs has been demonstrated various Zn‒O band positions at 485 cm^−1^ ^9^, 442 cm^−1^ ^29^, 400–500 cm^−1^ ^24^, 782 cm^−1^ ^30^, 450 cm^−1^ and 600 cm^−1^ ^12^, respectively, supporting biofabrication of present algal ZnO NPs. These results indicate that main organic contents of chlorella extract^31^ including proteins (45%), peptides (20%), fat (20%), carbohydrates (20%), vitamins (10%) and to some extent fiber (5%) were estimated to be dominant biomolecules involved in dual role both in Zinc ions (II) reduction and effective stabilization of fresh synthesized ZnO NPs. Moreover, it is anticipated that successful biofabrication of green ZnO NPs most likely carried out through the donor-acceptor mechanism as result of interaction between oxygen atoms of biofunctional groups (hydroxyl etc.) presence in chlorella extract and zinc ions of the salt precursor^32^ (Figs 4B, 5). As it is depicted in Fig. 5, OH groups in carbohydrate as biomolecule model would donate an electron to electrophile zinc species leading to oxidation of hydroxyl group and reduction of electron deficient Zn ions to Zn atoms. Generally, three stages including activation, growth and termination phase could involve in algae-assisted ZnO NPs biosynthesis process^33^. In an initial activation step, Zn (II) cations would extract from zinc nitrate salt precursor dissolved in water^7^. In presence of algae-derived biofunctional groups, Zn cations from divalent oxidation state would reduce to metallic form and during the air-drying process they immediately oxidize to ZnO NPs as result of the superior chemical reactivity of fresh bare nanoscale zinc metal surface. In growth and termination phases, the accumulation and stabilization of ZnO nanoparticles by metabolites of microalgae would occur, respectively. Comparison to parent algae extract, a decrease of O‒H peak intensity and the advent of C=O strong peak in biogenic ZnO NPs could reveal reduction–oxidation reaction between functional groups and Zn ions (Fig. 4B)^16^. This results are in compliance with several reports indicating biogenic synthesis of nanoparticles such as palladium nanocrystals^16^, and gold^34^ nanoparticles which indicated that diverse metabolites present in microalgae extracts such as polyphenols, sugars, terpenoids, proteins, phenolic acids, and enzymes are key responsible for reduction and stabilization of green nanoparticles^34,35^.

### SEM and TEM observations

**Figure 6:**
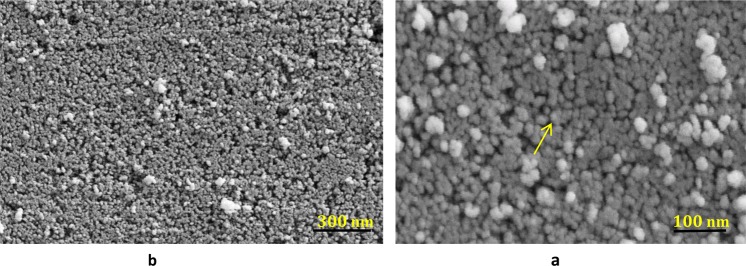
SEM images of (**a**) high magnification (40.0X and low magnification (15.23X) (**b**) of the biosynthesized ZnO NPs.

**Figure 7:**
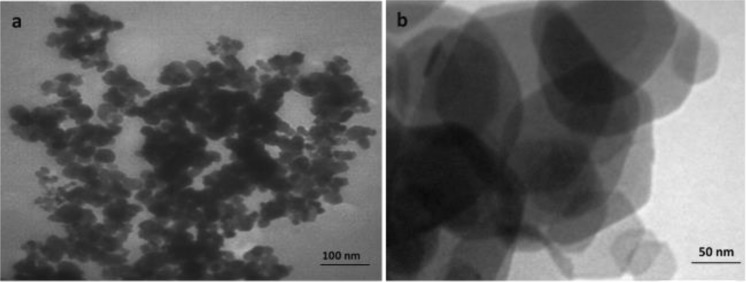
TEM images of (**a**) low magnification (35.97X) and high magnification (60.00X) (**b**) of the biosynthesized ZnO NPs.

Figure 6 is illustrated characteristic SEM images of chlorella microalgae-mediated biosynthesis of ZnO NPs. The nanoparticles were appropriately dispersed in the electron-rich biomolecules of the algal medium. From Fig. 6, it can be realized that proper morphological distribution of spherical ZnO NPs with a slight agglomeration and various size ranging from 20 to 50 nm were acquired in the optimized synthesis conditions. Apparently, the measured particle diameters in TEM and SEM techniques are fairly more appropriate methods compared to XRD patterns. XRD technique generally is employed for obtaining the crystal size and also dependents on particle shape while TEM and/or SEM imaging are used to obtain particle size which probably compromises polycrystalline^36^. Additional formation of particles organization, dispersion, and distribution of biogenic synthesized ZnO NPs was corroborated via TEM results indicating that nanoparticles are of hexagonal shape with the average size of about 20 ± 2.2 nm (Fig. 7). Similarly, XRD findings earlier confirmed the identical form (Hexagonal wurtzite structure) of produced ZnO NPs (Fig. 3). Eventually, TEM and SEM results evidently demonstrate that active organic compounds in the Chlorella microalgae template are possibly key responsible for zinc (II) cations reduction and capping of fresh formed ZnO NPs as well.

### Photocatalytic activity of ZnO NPs

**Figure 8:**
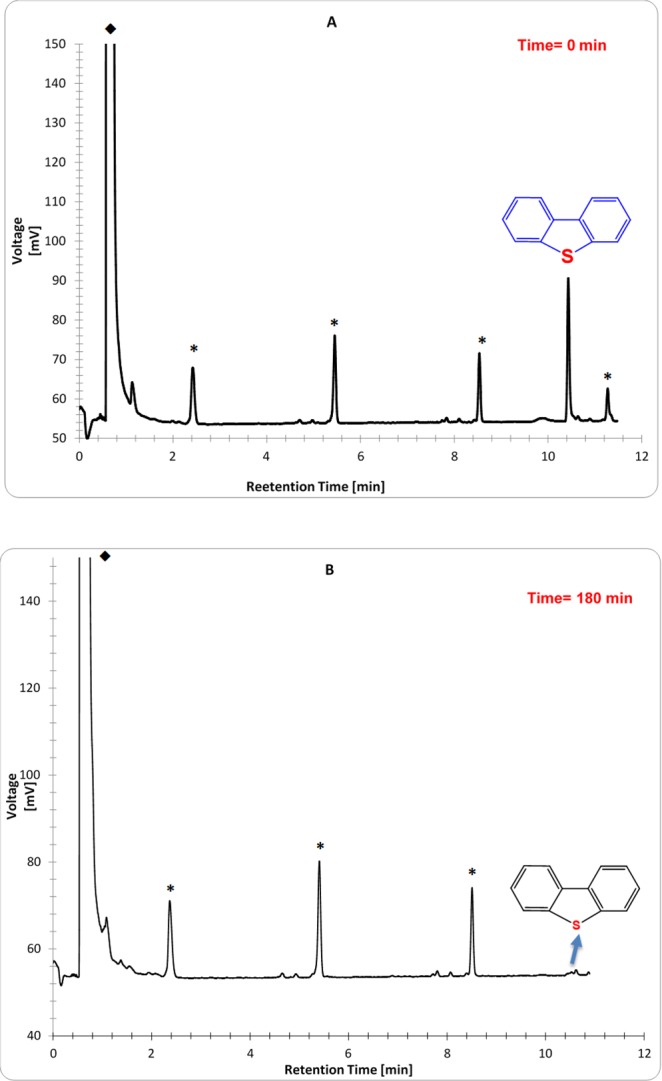
The GC chromatograms of DBT pollutant before (**A**) and after using green ZnO nanophotocatalyst (**B**); ✳ and ◆ signs refer to impurities and solvent, respectively.

**Figure 9:**
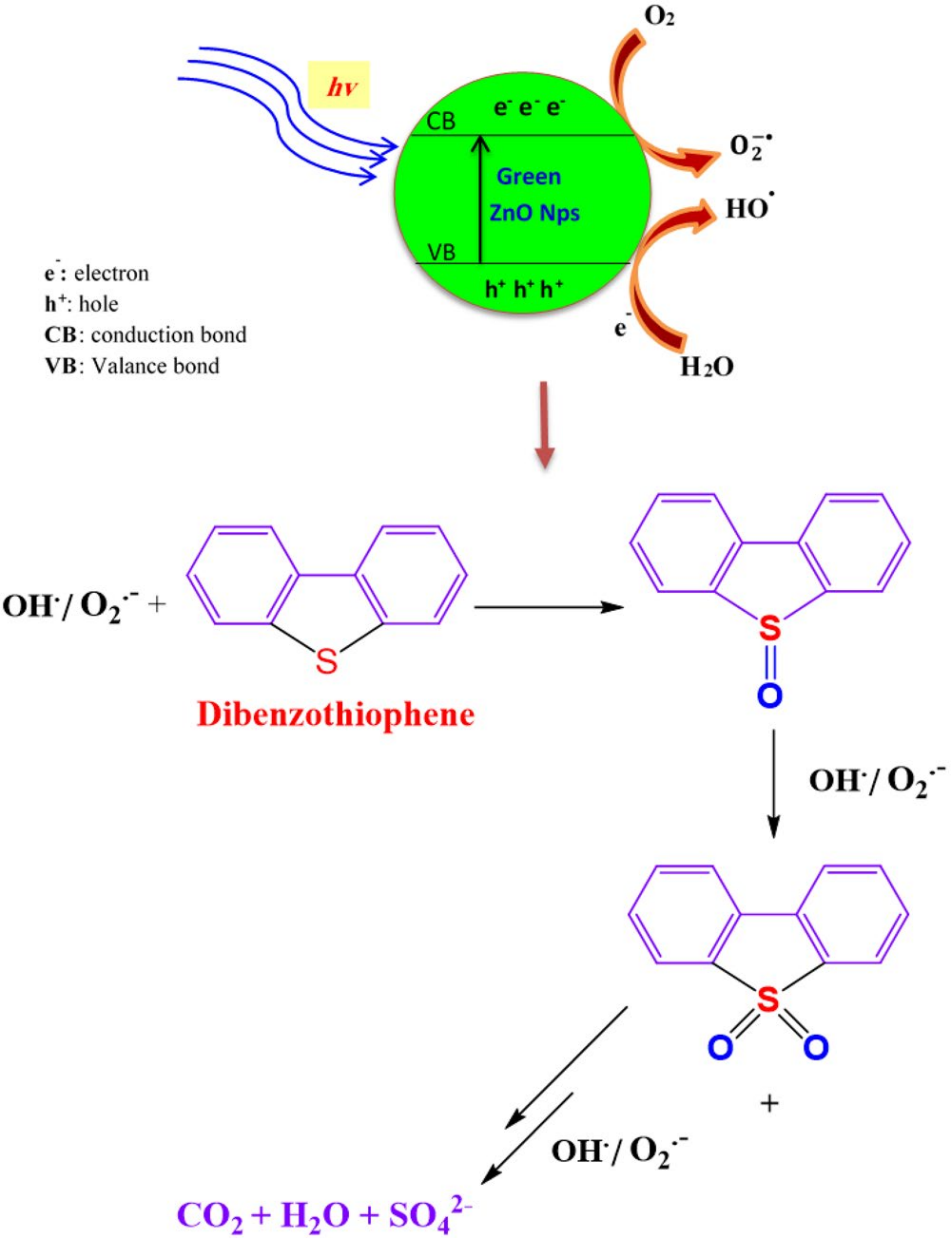
Proposed pathway for DBT photo-desulfurization using biosynthesized ZnO NPs.

To highlight the potential catalytic efficiency of green ZnO NPs toward environmental remediation and avoid the water pollution; photodegradation experiments of DBT aqueous solution was investigated under UV light irradiation in a homemade batch-type photoreactor which has been previously described in detail by our research group^37^. Under optimum preparation conditions (see Supplementary), typically 20 mL sample of wastewater comprising 0.01 g of nanophotocatalyst and 10 ppm of DBT with neutral pH of 7 was placed into the reactor. The solution was remained roughly for half an hour with constant stirring of 480 rpm at room temperature to reach the adsorption equilibrium. After UV light irradiation for interval times, the suspension is sampled and the degradation process of DBT was scrutinized through a gas chromatograph (GC). Each experiment was performed in triplicate so that confirms the repetition of DBT degradation process. The reduction of DBT concentration was determined in GC *via* comparing the retention time of DBT sample (Fig. 8A) prepared in laboratory^38^ and its final photocatalyzed solution by ZnO NPs. GC qualitative analysis was deduced that an extent of 97% of DBT pollutant was decomposed after 3 hours of photocatalytic reaction (Fig. 8B). The superior degradation efficiency of algal ZnO NPs was mainly triggered by its wider band gap value, which was determined to be 3.46 eV. Due to band gap broadening, the algal ZnO NPs highly likely absorb a considerable amount of UV light irradiation; which in turn increase the photodegradation rate of the DBT significantly. Likewise, Rawat *et al*. has been found that higher catalytic degradation of yellow dye (93.38%) was attributed to a wider band gap of biogenic ZnO NPs^30^. This result indicates that the lower load (0.01 g) of cost-effective and environmentally friendly ZnO NPs induce considerably higher catalytic performance toward DBT desulfurization compared to other reported catalysts. For instance, Khayyat and Roselin were indicated that H_2_O_2_/UV, Au/TiO_2_/UV, Au/TiO-H_2_O_2_/UV catalysts were degraded DBT to an extent of 9, 12.3, 46 and 61%, respectively^39^. Similarly, Li *et al*.^40^ have been demonstrated that DBT conversion of up to 84% was succeeded using Ru–Ni–Mo/Al_2_O_3_ catalyst under a rather difficult condition at a temperature of 380 °C for 11 h. Based on GC results, a provisional reaction mechanism of ZnO NPs function in the photocatalytic elimination of DBT is depicted in Fig. 9. Under UV illumination, electrons transference from valence bond (VB) to the conduction band (CB) in the irradiated ZnO semiconductor is led to electron-hole pair formation which in turn generates superoxide radical anion (•${{\rm{O}}}_{2}^{-}$) and hydroxyl radicals (•OH) in presence of H_2_O and O_2_ molecules^11^. These radical species, therefore, serve as reactive oxidizing agents in dynamic photocatalytic desulfurization of DBT (Fig. 9). The formation of sulfate ions was confirmed using barium chloride and dilute hydrochloric acid, where sulfate reacts with barium ions to form insoluble white barium sulfate precipitate. Meanwhile, the presence of carbon dioxide product was detected by titration method using standard NaOH solution^41^.

### The efficiency of the photocatalyst in a real sample

**Figure 10:**
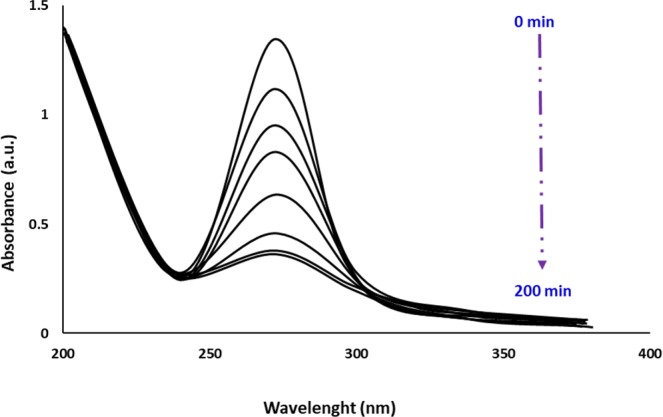
UV-vis spectra of photodegradation of DBT in real industrial wastewater using green ZnO NPs at different UV exposure time.

The performance of catalytic activity of algal ZnO NPs was examined also in the polluted environment. The real sample containing DBT was obtained from Abadan Oil refinery, Iran. Figure 10 illustrates the variation of photocatalytic degradations of diluted DBT solution (200 ppm) in presence of biological ZnO NPs (10 mg) at different times using UV-vis technique. It can be seen that the characteristic peak of DBT at 270 nm decreased gradually with increasing UV exposure time and almost disappeared after 190 min of photocatalytic reaction. The photocatalytic removal of DBT in the contaminated specimen was leveled off to an extent of 95% by algal ZnO NPs using Lambert Beer’s Law (Eq. 1)^30^.

1 $$R=({A}_{\circ }-A)/{A}_{\circ }\times 100$$

where A_0_ and A are contributed to the absorbance of the DBT at time t = 0 and t, respectively.

This results indicated that biosynthesized ZnO NPs demonstrated high photocatalytic efficiency toward the degradation of organic pollutants in wastewater.

### Reusability of biogenic ZnO Nps

**Figure 11:**
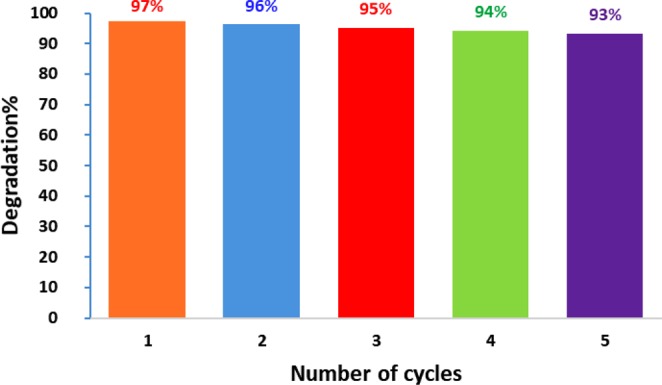
Reusability test of the photocatalytic degradation of DBT in five catalytic cycles using biological ZnO NPs.

To inspect the photostability and reusability of the biological ZnO Nps in the photodegradation of DBT, cycling experiments were studied using an oven drying method at 100 °C between reaction cycles^42^. After consecutive five cycles of photodecomposition, there is negligible change in the catalytic efficiency of the ZnO NPs. The degradation performance for the five runs was appeared to be slightly reduced from 97% to 93%, indicating higher durability and recyclability of biosynthesized ZnO NPs for degradation of DBT (Fig. 11). These findings are compatible with the results by Obendorf and Han^43^, Malghe and Lavand^44^, and Sun *et al*.^45^ for degradation of methyl parathion, Malachite green, and rhodamine B, respectively.

## Conclusions

In the present study, extracellular one-pot green synthesis of stable zinc oxide nanoparticles was successfully reported for the first time through electron-rich microalgae Chlorella extract as a reducing and stabilizing agent. Visual color change, UV-visible, FTIR, XRD, SEM and TEM techniques were all confirmed formation of highly pure and well dispersed ZnO NPs with average particle size around 20 nm. Under optimum experimental conditions, GC results revealed that as-obtained ZnO NPs demonstrate an excellent photocatalytic activity toward DBT photodegradation as a hazardous pollutant in the aquatic environment. In this study, synthesis of ZnO NPs in neutral aqueous solution at gentle temperature offers a facile, non-toxic, biocompatible and viable economic route for biological and environmental applications. The degradation rate of DBT continued almost constant after five cycle times showing higher stability and effectiveness of bio-assisted ZnO NPs. Due to the environmental benefits of biological biofabrication over conventional chemical and physical methods, further researches utilizing more potential natural sources for biogenic nanoparticles preparation are in progress.

## Materials and Methods

Zinc acetate (Zn(CH_3_COO)_2_·2H_2_O) (99.99%, w/w), Methanol (CH_3_OH), Ethanol (C_2_H_5_OH) and Dibenzothiophene (DBT) were purchased from Sigma-Aldrich (Missouri, USA). Deionized (DI) water was utilized for the preparation of all aqueous solutions and washing as well. Fresh crude microalgae Chlorella powder was provided from the Persian Gulf Science and Technology Park (Bushehr, Iran).

### Preparation of chlorella extract

For the preparation of microalgae Chlorella extract, the soaking method of extraction was utilized^46,47^. Typically, 1 gm of microalgae Chlorella powder was dispersed into 200 ml of DI water in the 250 mL Erlenmeyer flask by vigorous magnetic stirring for 15 minutes at a temperature of 80 °C using a stirrer-heater. Then the heated Chlorella extract was filtered through Millipore filter (0.1 um) and the collected supernatant was stored at 4 °C for further experiments.

### Preparation of zinc oxide nanoparticles

80 ml of zinc acetate solution was mixed with 20 mL of clean algal extract liquid at a temperature of 58 °C and retained for 60 minutes with continuous stirring at 150 rpm. Heating treatment was essential to accelerate the reduction process by the electron-rich organic biomolecules of microalgae Chlorella. The primary pH of the mixture solution was adjusted around 8 which in turn altered to 5.5 at the end of the reaction due to a decline of alkaline OH groups. The produced milky solution was stirred for an additional 25 min at 85 °C, and the resulting precipitate was centrifuged, washed with deionized water, and dried at 50 °C.

## Supplementary information

SUPPLEMENTARY
