# Supplementary material for: Phycosynthesis and Enhanced Photocatalytic Activity of Zinc Oxide Nanoparticles Toward Organosulfur Pollutants
Source: Sci Rep. 2019 May 3;9:6866. doi: 10.1038/s41598-019-43368-3 (PMC6499781; doi:10.1038/s41598-019-43368-3)
Supplement: Supplementary file 1 — SUPPLEMENTARY [file 41598_2019_43368_MOESM1_ESM.pdf]

Phycosynthesis and Enhanced Photocatalytic Activity of Zinc Oxide Nanoparticles Toward Organosulfur Pollutants

Taregh Khalafi, Foad Buazar*, Kamal Ghanemi

Department of Marine Chemistry, Khorramshahr University of Marine Science and Technology, P.O. Box 669, Khorramshahr, Iran

Table S1. Influence of time on elimination amount of DBT using green ZnO Nps

Time (min)	% Removal
15	34.75
30	35.35
45	38.95
60	42.60
75	53.05
105	75.85
120	85.42
180	97.00
200	87.75

* Corresponding author. Tel.: +98 9161150684; E-mail address: fb@kmsu.ac.ir, (F. Buazar).

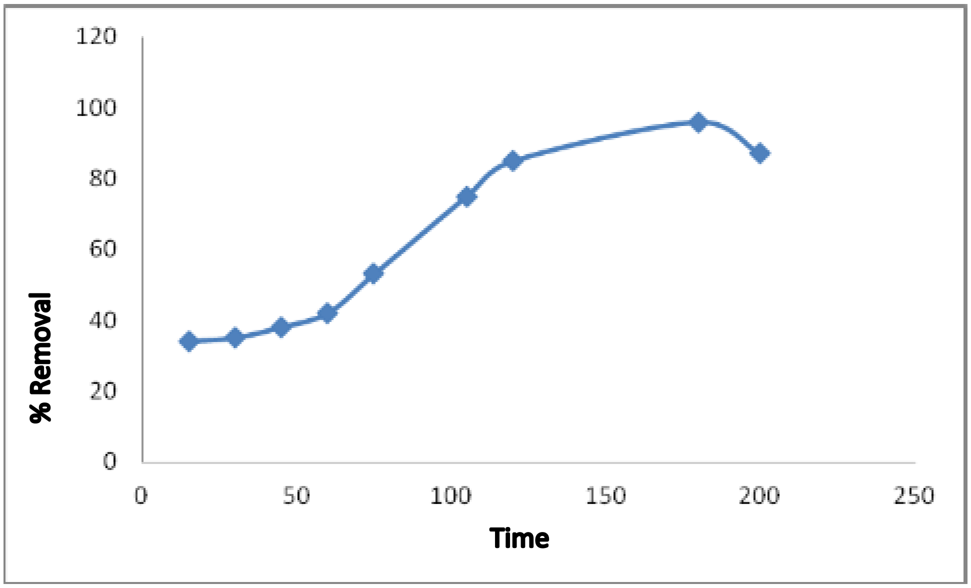

S1. Influence of contact time on elimination of DBT using green ZnO Nps

Table S2. Influence of concentrations of ZnO Nps on elimination amount of DBT .

ZnO Nps amount (g)	%Removal
0.01	95
0.05	90
0.1	60.88
0.15	55.97
0.2	46.79
0.25	45.01

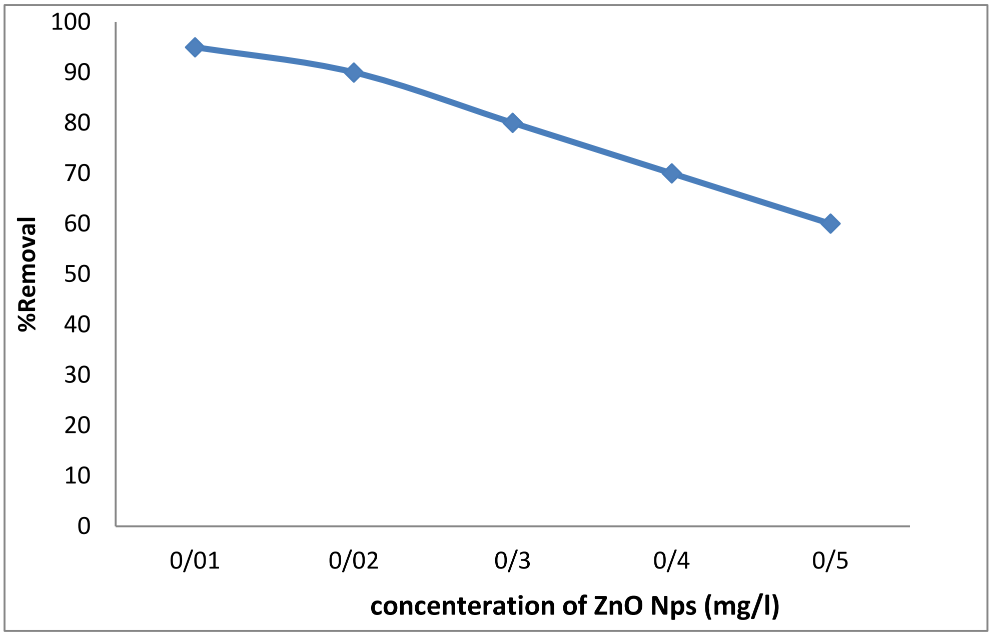

S2. Influnce of ZnO Nps concenteration on DBT degradation.

Table S3. Influence of concentration of solution on DBT removal.

Intitial concenteration (mg)	Removal
1	35.5
3	50.5
4	60.15
7	70.5
10	97
12	87.75

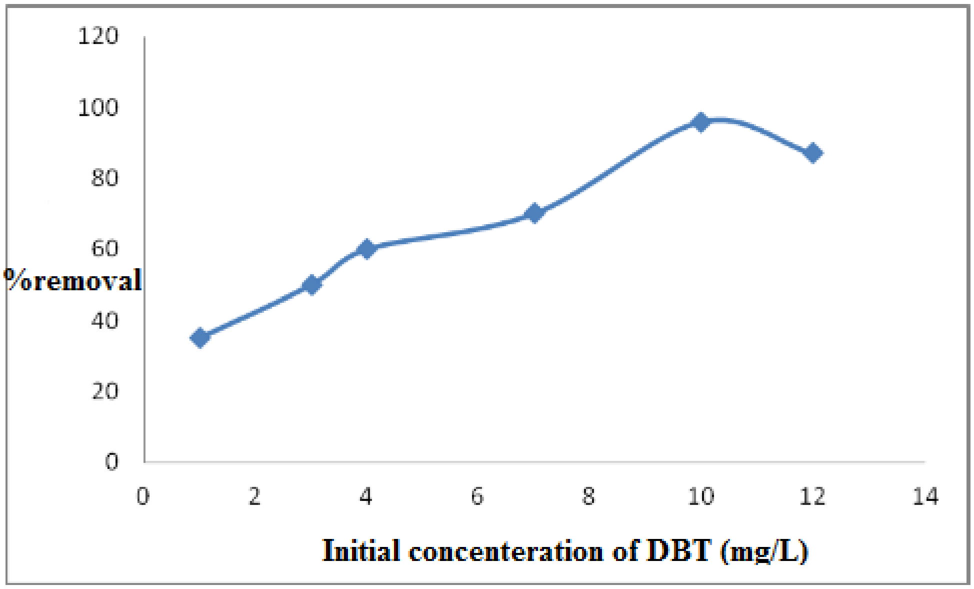

S3. Influnce of concenteration of initial solution on DBT removal .
